# Harm Minimisation Drug Policy Implementation Qualities: Their Efficacy with Australian Needle and Syringe Program Providers and People Who Inject Drugs

**DOI:** 10.3390/healthcare10050781

**Published:** 2022-04-22

**Authors:** Danielle Resiak, Elias Mpofu, Roderick Rothwell

**Affiliations:** 1 Faculty of Medicine and Health, School of Health Sciences, The University of Sydney, Sydney, NSW 2006, Australia; rod.rothwell@sydney.edu.au; 2 Rehabilitation and Health Services, University of North Texas, Denton, TX 76203, USA; 3 School of Human and Community Development, The University of the Witwatersrand, Johannesburg 2000, South Africa; 4 Family and Community Medicine, Meharry Medical College, Nashville, TN 37208, USA

**Keywords:** NSP, NSP service provider, PWID, implementation qualities, compatibility, observability, relative advantage, resourcing, trialability

## Abstract

(1) Background: Policies and laws in several jurisdictions across the globe have aimed to promote harm minimisation or reduction, through the implementation of Needle and Syringe Programs (NSP) for people who inject drugs (PWID), for whom abstinence may not be possible or desired. While NSPs hold great promise, their implementation qualities are understudied. (2) Aim: We aimed to examine the implementation quality priorities of NSP providers and PWID consumers in an Australian setting. (3) Method: This study utilised a Quantitative-qualitative (QUAN-qual) mixed methods approach. Survey participants included both PWID (*n* = 70) and NSP providers (*n* = 26) in Australia. (4) Results: Results following non-parametric data analysis indicate NSP providers prioritised NSP implementation qualities in the following order: compatibility, observability, relative advantage, resourcing and trialability. Contrary to which, PWID prioritised resourcing, compatibility, relative advantage and trialability, respectively. Findings demonstrate that efficacy of implementation qualities is dependent on the juxtaposition of service provision and utilisation whereby implementation quality priorities are balanced. (5) Conclusions: This research presents novel findings guiding NSP harm reduction programmes for sustainability framed on provider and consumer implementation quality priorities. We envisage future studies on boundary conditions of NSP harm reduction implementation in other jurisdictions.

## 1. Background

Drugs in circulation globally, have diversified over the past decade with an expansion of synthetic drugs and non-medical use of pharmaceutical and prescription medications, some of which carry high risk of dependency and addiction [1,2]. In recent decades, policies, and laws in several jurisdictions across the globe have aimed to promote harm minimisation or harm reduction (HR) from habit forming drugs inclusive of Australia [3], the United Kingdom [4], New Zealand [5], the Netherlands, Switzerland and some parts of the USA [6]. Harm reduction was initially developed in response to people who used drugs for whom abstinence was not feasible and has demonstrated effectiveness in the reduction of both morbidity and mortality in these populations [7]. It is a public-health approach to substance-abuse treatment based on reducing the negative consequences of drug use rather than eliminating drug use altogether [8]. Harm reduction encourages individual, community, policy, and environmental change through inter-sectoral and multi-level activities [9]. Harm reduction programmes include: (a) needle and syringe programmes (NSP); (b) opioid substitution therapies (OST) and (c) medically supervised injecting centres/drug consumption rooms [10]. Such well-established, user friendly and low threshold services have been developed through rapid and pragmatic community response [9]. Harm reduction approaches are community-friendly in proactively addressing risk for excess mortality in populations from otherwise preventable substance overdose and the unsafe use of addictive substances, in addition to minimizing the health cost through treating substance use disorders [11]. While NSPs hold great promise for the reduction of Blood Borne Virus (BBV) transmission [12], their implementation qualities are understudied. Implementation science aims to generate knowledge on evidence-based practices for the design of dependable interventions [13]. To achieve their purpose, harm reduction programmes should address the perceptions of providers and consumers. Thus, there is a particular need for implementation quality studies to determine what works for PWID and their NSP providers. This study aimed to address that gap in the evidence.

### 1.1. Implementation: Constructs and Applications 

Evidence-based implementation qualities included: relative advantage, compatibility, complexity, trialability, observability and resourcing [14]. Relative advantage refers to evidence of effectiveness to purpose compared to present practice. Compatibility refers to a match between the values, norms and needs of providers and consumers. Complexity refers to ease of implementation, to increase service access usage. Trialability refers to the option for non-committal use of a service type by providers and consumers, and observability refers to evidence of service implementation benefits. Lastly, resourcing refers to a programme’s capacity to meet the needs of consumers. Appropriately designed and implemented harm reduction programmes would have these implementation qualities, leading to reduced social costs and morbidity associated with drug dependence [15].

### 1.2. Implementation Priorities of NSP Service Providers

In theory, NSP implementation priorities would depend on policy and laws governing practices. For instance, in Australia, NSPs are guided by federal law, resulting in general consistencies yet not free of policy variation by jurisdiction [16]. Primary sites are operated by specialist staff skilled in brief interventions and referrals, while secondary NSP staff typically hold administrative roles within community/sexual health centres [17]. Additionally, secondary provider sites are limited by the range of injecting equipment provided [18].

Primary NSPs may be invested in exploring issues of the relative advantages of emerging versus current practices, the ease of implementation, opportunities to trial new service approaches, observability of outcomes and to customise services to consumer needs. Secondary NSPs may focus on complexity due to their competing service delivery requirements. Few jurisdictions have developed best practice recommendations and guidelines for NSPs [19,20]. Evidence from implementation science studies would be important for NSP guideline development.

### 1.3. Implementation Priorities of PWID

Despite the growing adoption of NSPs as a harm reduction strategy, PWID report barriers to safer injecting practices including stigma, fear of being exposed, limited NSP access and custody concerns [21,22]. For PWID, priority implementation qualities may include relative advantages compared to the existing or previous harm reduction services they have accessed, the ease of service access and the opportunity to trial a service for acceptability and compatibility to their needs [23], although evidence for which remains unclear. For instance, PWID may prioritise relative advantage based on factors of NSP accessibility. Such factors may include NSP geographic locations, transportation, hours of operation for service utilisation and policing practice [24,25,26]. Ease of access may be an overriding consideration for PWID who experience faster progress from initiation of drug use to dependence, such as those who identify as women [27], people with a history of incarceration [28] and those with a longer history of drug dependence [29]. Furthermore, there could be differences in implementation quality priorities between ethnic groups. For instance, Australian First Nations Peoples may prioritise compatibility, given that NSPs can lack First Nations Peoples’ staff [30].

### 1.4. The Australian NSP Implementation Context 

As previously noted, in Australia, NSPs are legally permitted through authorised services [31], providing injecting equipment as a harm reduction response to blood borne virus (BBV) transmission [21,32]. The first formal NSP operation in Australia was in NSW in 1986 [33]. With over 3000 NSP sites, Australia is estimated regularly to have one of the highest population level coverage rates globally [31,34]. NSPs in Australia consist of primary, secondary, mobile, outreach, pharmacy, and vending machine outlets [6].

**Figure 1:**
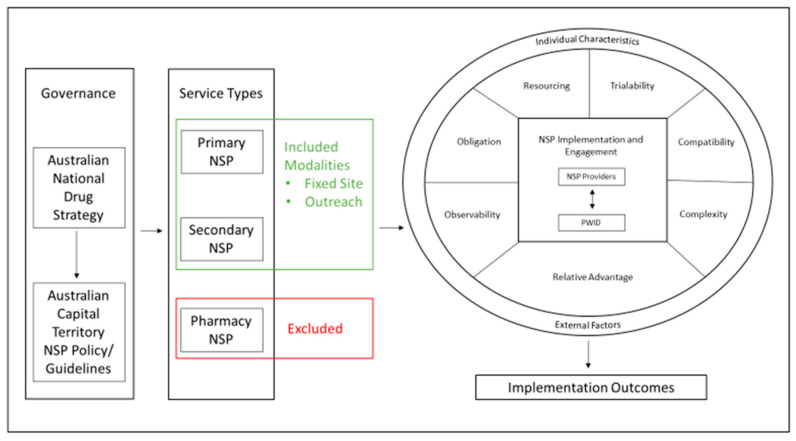
Conceptual Model.

Figure 1 presents a framework for understanding NSP implementation in an Australian setting as follows: (a) levels of influencing factors on implementation; (b) the context within which the policies, programmes and individual practice collectively known as the intervention takes place and (c) relationships between influential factors, and outcomes.

### 1.5. The Present Study

The present study aimed to profile the implementation quality priorities of the Australian NSP providers and the consumers PWID on the factors of relative advantage, observability, obligation, resourcing, trialability, compatibility and complexity. Our specific questions were:

1. What implementation qualities mattered more to the NSP providers and the consumers PWID?
2. How do implementation quality priorities differ among and between NSP and consumers PWID?
3. How are PWID socio-demographics associated with NSP implementation quality priorities?

To address these questions, we tested the following hypotheses and sub-hypotheses:

1. NSP providers prioritised programs’ relative advantage, trialability and observability of implementation qualities comparatively higher than PWID.
  - Primary NSP providers would prioritise compatibility and observability.
  - Secondary providers who may rate relative advantage, complexity and trialability implementation qualities higher.
2. PWID prioritise compatibility and trialability higher than their NSP providers.
  - PWID with previous engagement in drug treatment and or HIV/HCV screening would prioritise observability and trialability.
  - PWID with previous engagement in the criminal justice system would prioritise resourcing, compatibility, and relative advantage.

Greater NSP access at a reduced cost, customisable to the needs of PWID, are some of the downstream health, social and economic benefits of collaborative NSP implementation [35]. Identifying service user (PWID) implementation priorities allows for NSP service provision to be implemented and evaluated for customisation.

## 2. Method

### 2.1. Research Design

This study utilised a Quantitative-qualitative (QUAN-qual) mixed methods’ approach [36] to characterise implementation quality priorities by NSP providers and their clients, PWID. The QUAN-qual method refers to the predominant QUAN measures which are supported to a lesser extent by qual measures to provide context to the QUAN findings. Thus, the QUAN-qual approach has the advantage of strengthening the credibility of the QUAN findings through broadening the scope of exploration beyond parameters initially imposed by the QUAN measures.

### 2.2. Participants and Setting

Survey participants were from 10 NSP sites in the Australian Capital Territory (ACT), Canberra. The ACT is host to 2 primary NSPs, 8 secondary NSPs, over 30 pharmacy NSP outlets and 6 syringe vending machines [18]. Our sample of participants comprised of 26 service providers and 70 PWID (see Table 1 for participants’ demographics). Primary and Secondary NSPs, excluding pharmacy outlets licensed to provide sterile injecting equipment to PWID, were approached to participate in the study. Employed or volunteer NSP provider participants were 18 years or above with at least 1 years’ experience. New NSP staff in training or with less than a year’s experience were excluded from the study. People Who Inject Drugs were included if they were aged 18 or above and collected equipment from an NSP in Canberra ACT.

### 2.3. Measures and Data Collection

Participants self-reported their socio-demographics (see Table 1) and completed a Service Provider Participant Questionnaire (SPPQ) if the participant was an NSP provider, or a Service User Participant Questionnaire (SUPQ) if the participant was a PWID [35]. A subsample of participants (*n* = 12) completed the focus group discussion on implementation quality priorities, as described below.

#### 2.3.1. The Service Provider Participant Questionnaire (SPPQ)

Service providers’ implementation qualities. The SPPQ is a 17 item measure of implementation qualities of trialability (confidence, strategy implementation and NSP guidelines, *n* = 3), compatibility (accommodate needs, personalised service and referral protocol, *n* = 4), relative advantage (communication, understanding NSP, NSP evaluation discussion and enhanced quality of life, *n* = 4), observability (NSP goals, proportion written, monitoring methods, evaluation process and implementation preparedness; *n* = 5), and resourcing (resources, external contributions, NSP funding, sharing resources and requirements, *n* = 5). Items are scored Yes/No (1, 0) or True/Not True (1,0).

Sample trialability questions were *“How confident do you feel in your ability to carry out the required duties of a Needle and Syringe Program service provider?”. “I have analysed the relevant Needle and Syringe Program Guidelines, so I am better equipped to implement the program as it was intended”* (Cronbach’s α = 0.71). Sample *relative advantage* questions were *“I have discussed with others the ways this Needle and Syringe program could better enhance the health-related quality of life of clients”* and *“I have participated in evaluation discussions regarding the implementation of this Needle and Syringe program”* (Cronbach’s α = 0.67). Sample compatibility questions included *“At this Needle and Syringe Program, we accommodate the needs of our clients according to their social and cultural background”* and *“We personalise the service provided to meet the needs of each person who injects drugs”* (Cronbach’s α = 0.88).

Sample *observability* questions included *“I feel sufficiently prepared to implement the Needle and Syringe program effectively”* and *“Methods for monitoring and evaluating the successful implementation of the services provided at this Needle and Syringe program are in place”* (Cronbach’s α = 0.71). Sample *resourcing* questions included *“Is there funding for the program?”* and *“I have discussed with others the sharing of resources in order to implement the Needle and Syringe program more effectively”* (Cronbach’s α = 0.52). We observed a high Cronbach’s α = 0.86 for the full SPPQ scale. The Cronbach’s alpha values we observed were satisfactory to high, suggesting reliability of scores for research purposes [37].

#### 2.3.2. The Service User Participant Questionnaire (SUPQ)

PWID implementation quality measures. The SUPQ comprised 15 items trialability (NSP drug treatment, Access of NSP treatments; *n* = 4), compatibility (operational hours, sociocultural needs and personalisation; *n* = 3), relative advantage (service provision, unwanted interventions, attendance benefits and community health, *n* = 4) and resourcing (seeking help, free equipment, identifying NSP and attendance, *n* = 4). Sample trialability questions included *“Undergone treatment or therapy for drug use previously?”* and *“If no, what is the main reason for not undergoing treatment or therapy for drug use?”* (Cronbach’s α = 0.61). Sample compatibility questions included *“Have the hours of operation of your preferred Needle and Syringe Program been suitable for your needs?”* and *“Do you feel as though staff personalise the service provided to meet your needs?”* (Cronbach’s α = 0.51). Sample relative advantage questions included *“Attending my local Needle and Syringe Program is beneficial to my health and wellbeing”* and *“My local community-based Needle and Syringe Program also benefits the health and wellbeing of my community members who don’t inject drugs”* (Cronbach’s α = 0.53). Sample resourcing subscale questions included *“Has the injecting equipment provided at your preferred Needle and Syringe Program always been provided free of charge?”* and *“My local NSP is a place in which I feel comfortable asking for help if I ever need it”* (Cronbach’s α = 0.73). The reliability of scores from the full UPQ scale achieved a high Cronbach’s α of 0.77.

#### 2.3.3. Open Ended Questions

Both NSP staff and PWID responded to open-ended questions to clarify responses to questions within sections of the survey. In addition, the PWID completed a focus group discussion on the use of NSPs within community and state service facilities, availability of sterile injecting equipment, access to specialised equipment needed to inject the substance of choice, who collects sterile injecting equipment and what an ideal NSP would resemble. For data trustworthiness, we had a strong research partnership with the PWID and their NSPs [35], and used reflexivity [38], as an additional method to establish data trustworthiness. This occurred through the experiential reflections and emerging awareness of any assumptions or biases in addition to peer debriefs

#### 2.3.4. Procedure

The Ethics Review Committee (RPAH Zone) of the Sydney Local Health District approved the study (X17-0175 & HREC/17/RPAH/256). The conduct of this study at ACT Health sites was authorised by the ACT Health Research Ethics and Governance Office (ETH.6.18.101E). Participants consented to the study in writing. We informed them of the study goals and the voluntary nature of the study, as well as their right to discontinue their participation from the study at any time without penalty. In addition, we assured participants of the confidentiality and anonymity of their data. Participants completed the survey at their NSP service centres. Similarly, the focus group discussion with PWID was hosted at a peer secondary NSP. As a token of appreciation, survey participants were offered a chocolate.

## 3. Data Analysis

### User and Provider Implementation Priority Ratings

We utilised IBM SPSS Statistics 25 for the survey data analysis. First, we weighted the scores by a factor of 1 for better differentiation of the scores on the decimal mean scale (0.00–0.99). Specifically, we employed non-parametric analysis to profile the implementation quality priorities of NSP and PWID, performing within group analysis (with NSP providers, and with PWID). Non-parametric tests are distribution free and with no risk for violation of assumptions as with parametric tests [39,40]. They are especially appropriate with small sample sizes from unique study populations for which there are limited baseline data on distributional qualities [41]. In doing so, we principally utilised the Wilcoxon Signed Rank Test, Mann–Whitney U and the Kruskal–Wallis to compare the relative importance of implementation qualities between NSP and within PWID by service demographics and personal factors, respectively. We report preference effect sizes using Cohen [42] of d = 0.2 for small effect size, d = 0.5 for medium effect size and d = 0.8 for large effect size. In addition, we controlled for possible inflation of Type 1 error by testing all hypothesis at the 95th confidence level (.05). We utilised the qualitative data to clarify the meanings from the quantitative analysis, providing a context for the findings [43].

## 4. Results

### 4.1. Descriptive Statistics

Table 2 presents correlation descriptive statistics for the implementation qualities for both the service providers and the PWID participant groups. For the NSP provider group significant positive correlations were observed between trialability and compatibility r(36) = 0.803, *p* = 0.002, compatibility and resourcing r(38) = 0.656, *p* = 0.015, compatibility and observability r(25) = 0.877, *p* = 0.002, relative advantage and resourcing r(50) = 0.487, *p* = 0.012, trialability and resourcing r(48) = 0.496, *p* = 0.014 and trialability and complexity and relative advantage r(48) = 0.479, *p* = 0.018.

For the PWID participant group, positive correlations were observed for compatibility and resourcing r (123) = 0.666, *p* = 0.000, relative advantage and resourcing r (119) = 0.740, *p* = 0.000 and relative advantage and compatibility r (120) = 0.653, *p* = 0.000.

### 4.2. NSP Implementation Quality Priorities 

**Figure 2:**
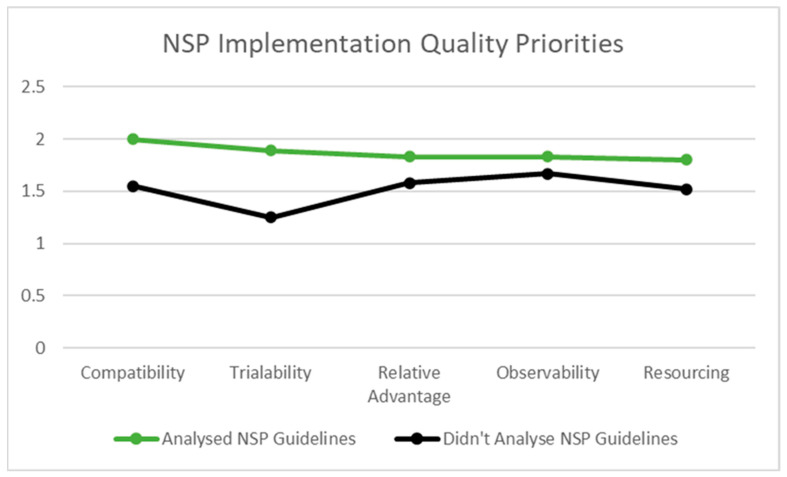
Implementation Quality Priorities of NSP Providers Who Have and Have not Analysed NSP Guidelines.

Table 3 and Figure 2 present the results for the implementation priority ratings by NPS providers and PWID. As can be seen from Table 3, NSP providers prioritised NSP implementation qualities in the following order: compatibility, observability, relative advantage, resourcing and trialability. Overall, NSP providers prioritised compatibility (Mean = 1.83, SD = 0.31) significantly greater than trialability (Mean = 1.57, SD = 0.40) T = 21, *p* = 0.026, *r* = 0.46. Likewise, compatibility (Mean = 1.83, SD = 0.31) was prioritised significantly more than resourcing (Mean = 1.66, SD = 0.21) T = 10, *p* = 0.043, *r* = −0.40. When split by service type (Primary versus Secondary NSP) a significant difference between ratings of trialability and compatibility was reported by secondary NSP providers T = 21, *p* = 0.026, *r* = 0.67. No significant difference between ratings of trialability and compatibility were reported among primary NSP providers.

### 4.3. Contextual Influences on Implementation Quality Priorities 

#### 4.3.1. NSP Guidelines

NSP providers who reported having analysed relevant NSP guidelines, to be better equipped to implement NSPs as intended, prioritised compatibility (Mean = 2; SD = 0.00), trialability (Mean = 1.89, SD = 0.22), relative advantage (Mean = 1.83, SD = 0.19), observability (Mean = 1.83, SD = 0.20) and resourcing (Mean = 1.80, SD = 0.14) higher than those NSP providers who had not analysed NSP guidelines; compatibility (Mean = 1.55, SD = 0.37), trialability (Mean = 1.25, SD = 0.25), relative advantage (Mean = 1.58, SD = 0.28), observability (Mean = 1.67, SD = 0.33) and resourcing (Mean = 1.52, SD = 0.17), respectively. See also Figure 2.

In each case, secondary NSP providers who reported having reviewed NSP guidelines to implement the programme as intended rated trialability *U* = 77.000, *z* = 3.344, *p* = 0.001, *r* = 0.79, compatibility *U* = 15.000, *z* = 2.582, *p* = 0.036, *r* = 0.91 and resourcing *U* = 77.000, *z* = 2.724, *p* = 0.008, *r* = 0.62 implementation qualities higher than the secondary NSP providers who had not reviewed the NSP guidelines. Furthermore, secondary NSP providers who reported confidence in their NSP service provision ability rated trialability *U* = 77.000, *z* = 3.344, *p* = 0.001, *r* = 0.79 and relative advantage *U* = 70.000, *z* = 2.174, *p* = 0.043, *r* = 0.50 significantly higher than those who were less confident.

#### 4.3.2. Evaluation Process and Funding

NSP providers’ ratings of observability differed significantly between NSP providers who reported having an evaluation process (Mean = 2.00, SD = 0.00) compared to those who reported not having an evaluation process or being unsure if there was one (Mean = 1.62, SD = 0.23), respectively, U = 45.00, *z* = 3.15, *p* = 0.001, *r* = 0.84. NSP providers engaged in evaluation processes reported, *“Statistics are kept regarding service delivery plus we have a process for clients to provide feedback regarding the service we provide”.* Furthermore, prioritising observability was identified as being built into NSP policy and procedure, “*We are governed by relevant policies and procedures and undertake monthly data analysis, monthly trend analysis and generate six monthly performance reports*”. On the contrary, the lower priority ratings of observability reported by the NSP provider participants may be due to a lack of knowledge of, and/or involvement in, evaluation processes whereby complacency in service provision and NSP implementation methods are challenged. NSP providers reported statements inclusive of, “*Unsure*”, “*I have no idea?*” and “*Unsure. Secondary program and not part of this process*”. These data suggest that when NSP providers at the very least have knowledge of an evaluation process, they are more likely to prioritise observability due to the reflective practice that leads to observable outcomes.

NSP providers who reported programme funding rated observability (Mean = 1.91, SD = 0.11) and resourcing (Mean = 1.89, SD = 0.16) implementation qualities higher than those who reported not having funding, observability (Mean = 1.60, SD = 0.28) and resourcing (Mean = 1.58, SD = 0.16).

Table 3 presents the results of the analysis by the contextual factors for NSP providers and PWID, applying the Mann–Whitney U and Kruskal–Wallis, respectively.

#### 4.3.3. PWID Implementation Quality Priorities

As shown in Table 3, PWID prioritised NSP implementation qualities in the following order: resourcing (Mean = 1.94, SD = 0.17), compatibility (Mean = 1.88, SD = 0.23), relative advantage (Mean = 1.87, SD = 0.18) and trialability (Mean = 1.64, SD = 0.24). Overall, PWID prioritised resourcing significantly higher than compatibility T = 3, *p* = 0.013, *r* = −0.32 and relative advantage T = 5, *p* = 0.001, *r* = −0.42. Trialability was rated significantly lower than resourcing among PWID T = 2, *p* = 0.001, *r* = −0.72. Focus group discussion comments validated this finding with PWID reporting several resourcing issues inclusive of location, stating, “*Yeah, but they can go to places and get help and everything but when you go out into these remote areas they just, there’s nothing there for em”.* Number of fits that could be provided, “*You must remember that all health centres, you got Belconnen, Gungahlin health centre, you got Belconnen, Phillip, Civic, ACT health centres that you can go up to the foyer and their little side, you walk up to the side of the counter and say, that’s where you go and ask for your fit packs but you can’t get a bulk of 100 you can only get a 5 pack or 3 pack”.* Additionally, the operational hours and cost were not always compatible to consumer needs, *“Weekends they’re not open and if you don’t have $2, you’re not going to get one”.*

#### 4.3.4. Drug Treatment Experience

There was a statistically significant difference in ratings of trialability between PWID who had versus those who had not previously engaged in drug treatment *H*(1) = 4.157, *p* = 0.041. PWID who had previously engaged in drug treatment rated trialability higher (Mean = 1.87, SD = 0.12) than PWID who have not previously been engaged in drug treatment (Mean = 1.60, SD = 0.24). There was a statistically significant difference in ratings of trialability between PWID who felt they did not have a need for drug treatment compared to PWID who reported a difficulty in accessing drug treatment *H*(1) = 8.407, *p* = 0.004. Trialability was rated higher among PWID who did not feel they had a need for drug treatment (Mean = 1.75, SD = 0.15) compared to PWID who reported a difficulty in accessing drug treatment (Mean = 1.45, SD = 0.26).

#### 4.3.5. HIV and/or HCV Test

There was a statistically significant difference in ratings of trialability between PWID who had previously been tested for HIV compared to PWID who had not had a prior HIV test *H*(1) = 7.784, *p* = 0.005. Those PWID who reported having been previously tested for HIV rated trialability higher (Mean = 1.71, SD = 0.16) than PWID who reported having not previously been tested for HIV (Mean = 1.20, SD = 0.20).

#### 4.3.6. History of Incarceration

For those PWID who reported not having self-injected while in prison there was a statistically significant difference in the ratings of resourcing (Mean 1.92, SD 0.22), and compatibility (Mean 1.90, SD 0.25) across genders *H*(1) = 6.000, *p* = 0.014. Males ranked both resourcing (Mean rank = 7.00) and compatibility (Mean rank = 7.46) implementation qualities significantly higher than female participants (Mean rank = 1.00 and 1.50), respectively.

For service user participants (PWID), who reported self-injection while in prison, a statistically significant difference in ratings of relative advantage were observed across gender *H*(1) = 4.213, *p* = 0.040. In this group males (Mean rank = 8.86) rated relative advantage significantly higher than females (Mean rank = 4.83).

## 5. Discussion

Both primary and secondary NSP providers ranked NSP implementation qualities in the following order: compatibility, observability, relative advantage, resourcing and trialability, yet the weighting of priority scores differed between each service type group. The higher weighting of implementing qualities by primary NSP providers may be explained by documented differences in staffing demographics. Primary sites are generally operated by specialist staff with a singular focus on harm reduction while secondary NSP staff typically hold administrative roles within community or sexual health centres [17]. Additionally, secondary NSP providers have a broader range of tasks and clientele to triage, which could influence their relatively lower rating of implementation qualities as compared to the primary sites [17]. Therefore, the implementation quality ratings are likely sensitive to broader competing tasks not experienced in primary NSP contexts, as demonstrated by the equity of implementation quality ratings among primary NSP participants.

Regardless of the NSP service type, NSP providers familiar with NSP guidelines prioritised compatibility, trialability, relative advantage, observability, and resourcing higher than the NSP providers who had not analysed them. The use of NSP guidelines provided benchmarks for programme evaluation and aid in identifying targets for improvement at both the individual programme and systems levels [19]. NSPs need to continuously adapt their harm reduction strategies to optimally target successive cohorts of PWID [44]. A significant difference in observability priorities among NSP providers who reported an evaluation process is likely due to their involvement in evaluating NSP effectiveness and noted opportunities for adaption leading to observational differences in service provision. Furthermore, observability is an inbuilt implementation quality for NSPs, particularly for those trying to demonstrate both a need for, and capacity to, deliver [45].

NSP providers who reported programme funding rated observability and resourcing implementation qualities higher than those who reported not having funding. Financial resources such as programme funding minimises the risk for rapid depletion of available resources [46]. If NSP services are funded, they are likely to recognise the benefit of funding through observable outcomes. Furthermore, Strike, Watson, Lavigne, Hopkins, Shore, Young, Leonard and Millson [19] point out, if supplies come at no cost to providers, NSPs are more likely to distribute them according to recommendations.

Secondary NSP providers who reported confidence in their NSP service provision ability rated trialability and relative advantage significantly higher than those who were less confident. This would be important in secondary sites, where NSP providers are typically customer service officers responsible for varied tasks beyond NSP provision [47]. With competing work demands, NSP provider confidence is likely to mitigate some workflow barriers to NSP implementation typically experienced at secondary NSP services [48,49].

The sociodemographic of PWID substantially explained their implementation quality priorities. For instance, PWID with previous engagement in drug treatment or HIV/HCV testing prioritised trialability higher than PWID who had not. Furthermore, PWID who reported not having a need for drug treatment prioritised trialability higher than PWID who reported a difficulty in accessing treatment. Findings may be variously explained. Firstly, society stigmatises PWID [50]. The social processes of stigmatisation can affect services due to stigma ascribed to individuals or groups becoming embodied by the places they frequent [45]. Conversely, stigma attributed to a service can result in the stigmatisation of those who frequent the service [45]. The willingness to trial NSPs may in part be due to trust, which in therapeutic encounters has been shown to facilitate a willingness to seek care, alter behaviour, encourage service usage and both uptake and adherence to treatment [51]. Secondly, many people who frequent NSP programmes are HCV positive [52]. Previous work has highlighted the utilisation of NSPs association between HCV prevalence and transmission [12]. NSPs are uniquely positioned to provide linkage to testing and treatment services [47]. Access to conventional health services for PWID is limited by a range of complex barriers, which low threshold services such as NSPs mitigate [53].

Relative advantage was rated significantly higher by male participants who reported both a previous incarceration and having self-injected while in prison. Albeit at a lower rate than in the community, injecting drug use continues to occur in prison [54], and Cunningham, et al. [55] report that for prisoners with a history of injecting drug use, between one third and three quarters will continue to inject in prison. This is consistent with our sample, having 45.5% of PWID reporting self-injecting while in prison.

### 5.1. Implications for Harm Minimisation Policy and Practice

The sustainability of NSPs is dependent upon both accessibility and continued service utilisation by the population base it aims to serve PWID [26]. However, despite the recognised need to monitor harm reduction programmes, comprehensive regularly updated systems for collating, critiquing and synthesising data on such is lacking [56]. Likewise, the generation of knowledge on NSP implementation practices for the design of dependable interventions is seldom addressed [13]. As reported in Haines et al. [57] and Strike, Watson, Lavigne, Hopkins, Shore, Young, Leonard and Millson [19], facilitators and barriers to guideline uptake related to health services exist at practitioner, political environment and healthcare system levels. Findings highlighted how guidelines provide benchmarks for programme evaluation and the significant difference in observability priorities among NSP providers who reported evaluation processes. Observing emerging drug use allows clear public health messaging to be developed [58]. Suggesting NSP guidelines, informed by the implementation quality priorities of providers and consumers, may enhance NSPs ongoing reinvention to suit successive cohorts of PWID [44].

### 5.2. Limitations and Suggestions for Further Research 

We note the following study limitations. First, this study was conducted among a Canberra ACT population of NSP service providers and PWID consumers. NSP implementation quality priorities may vary across other Australian or international jurisdictions. Second, analysis of NSP provider data was limited by sample size, preventing a within groups analysis of primary NSP providers. Third, this study was cross sectional in design, which does not allow for causal inference. Fourth, the study relied on self-report measures which are susceptible to social desirability biases compared to direct observational data. Future studies should seek to recruit a probability and longitudinal design supplemented with observational data for more conclusive findings.

## 6. Conclusions

This research presents novel findings as to the implementation quality priorities of Australian NSP providers and PWID consumers. NSP providers prioritised NSP implementation qualities in the following order: compatibility; observability; relative advantage; resourcing and trialability. Contrary to which, PWID prioritised the following: resourcing; compatibility; relative advantage and trialability, respectively. The differences in implementation quality priority ratings were explained by contextual and sociodemographic influences. Harm reduction programmes should consider aspects of compatibility that are important to NSP providers for sustainable service provision, coupled with the resourcing priorities of PWID. Doing so is important for increasing the likelihood of service utilisation and in turn reducing risks associated with injecting drug use. Findings demonstrate that the efficacy of implementation qualities is dependent on the juxtaposition of service provision and utilisation whereby implementation quality priorities are balanced to achieve intended individual and community level harm reduction.

## Figures and Tables

**Table 1:** Demographics of PWID and NSP Provider Participants.

Demographics	PWID		NSP Providers	
	*n*	%	*n*	%
**Gender**				
Male	43	60.6%	8	30.8
Female	26	36.6%	18	69.2
Other	-	-	-	-
**Sexual Identity**				
Heterosexual	49	69%		
Bisexual	11	15.5%		
Homosexual	4	5.6%		
**Ethnicity**				
Aboriginal or Torres Strait Islander	11	15.5%	1	3.8%
Others	56	78.9%	25	96.2%
**Age of First Injection**				
18–24	27	38%	1	3.8%
25–31	10	14.1%	-	-
37–43	8	11.3%	2	7.7%
Other	24	33.8%	1	3.8%
Have never self-injected	-	-	22	84.6%
**Age Range**				
18–30			7	26.9%
31–40			3	11.5%
41–50			5	19.2%
51 and above			11	42.3%
**Previous Imprisonment**				
Yes (1)	33	46.5%		
No (0)	36	50.7%		
**Highest Academic Qualification**				
Self-Reported				
**Highest Professional Qualification**				
Self-Reported				
**Years in Current Profession**				
1–2 years			10	38.5%
3–4 years			-	-
5–6 years			3	11.5%
Above 6 years (specify how many)			12	46.2%
**Other Professional Qualification**				
Self-Reported				
**NSP Sector**				
Public			10	38.5%
Private			*-*	*-*
Non-profit organisation			13	50%
Other			2	7.7%
**Number of NSPs You Have Worked For**				
1			25	96.2%
**Self-Injection in Prison**				
Yes	15	21.1%		
No	52	73.2%		
**Substance of Choice**				
Methamphetamine	22	31%		
Cocaine	-	-		
Heroin	29	40.8%		
Methadone	5	7%		
Multiple	13	18.3%		
Another drug-unspecified	1	1.4%		
**Frequency of Self-Injection**				
Multiple times a day	16	22.5%		
Once daily	16	22.5%		
Multiple times a week but not daily	18	25.4%		
Once a week	12	16.9%		
Multiple times a month but not weekly	5	7%		
Once a month	2	2.8%		
Less than once a month	2	2.8%		
**Overdose in Previous 12 Months**				
Yes	13	18.3%		
No	57	80.3%		
**Frequency of Sterile Equipment Use**				
For all self-injections	53	74.6%		
Most of the time	17	23.9%		
**History of Drug Use Treatment**				
Yes	45	63.4%		
No	23	32.4%		
**Previous HIV Test**				
Yes, within the last year	42	59.2%		
Yes, prior to last year	19	26.8%		
Never	8	11.3%		
**Previous HCV Test**				
Yes, within the last year	46	64.8%		
Yes, prior to last year	13	18.3%		
Never	8	11.3%		
**HCV Treatment**				
Antiviral treatment	18	25.4%		
No antiviral treatment	44	62%		

**Table 2:** NSP Provider and PWID Implementation Quality Correlations.

	M	SD	1	2	3	4	5
**NSP Provider**							
1. Resourcing	1.66	0.21					
2. Observability	1.76	0.26	0.502				
3. Compatibility	1.83	0.31	0.656 *	0.877 *			
4. Relative Advantage	1.70	0.26	0.487 *	0.344	0.533		
5. Trialability	1.57	0.40	0.496 *	0.290	0.803 **	0.479 *	
6. NSP Type	1.85	0.37	−0.287	−0.357	−0.246	−0.284	−0.202
7. NSP Guidelines	1.50	0.51	0.673 **	0.310	0.729 **	0.481 *	0.818 **
8. NSP Funding	1.27	0.45	0.661 **	0.622 *	0.384	0.029	0.144
9. Evaluation Process	1.23	0.43	0.368	0.716 **	0.324	0.365	0.215
**PWID**							
1. Resourcing	1.94	.17					
2. Observability	-	-	-	-	-	-	-
3. Compatibility	1.88	0.23	0.666 **				
4. Relative Advantage	1.87	0.18	0.740 **	-	0.653 **		
5. Trialability	1.64	0.24	0.101	-	0.327	0.247	
6. Drug Treatment	1.66	0.48	0.136	-	0.117	0.123	0.395
7. Reason for Not	1.64	0.49	0.095	-	0.441*	0.260	0.625 **
8. HIV Test	1.88	0.32	0.076	-	0.110	0.048	0.758 **
9. HCV Test	1.88	0.33	0.048	-	0.232	0.123	0.758 **

** Correlation is significant at the 0.01 level (two-tailed). * Correlation is significant at the 0.05 level (two-tailed).

**Table 3:** Analysis of implementation quality priorities within NSP providers and PWID groups.

Implementation Qualities		NSP Provider							PWID							
		NSP Guidelines		NSP Funding		NSP Evaluation Process			Drug Treatment		Reason for Not		HIV Test		HCV Test	
		Yes	No	Yes	No	Yes	No		Yes	No	Don’t feel a need for it.	Difficulty, no referral or other.	Yes	No	Yes	No
Trialability	M	1.89	1.25	1.67	1.54	1.73	1.53	M	1.87	1.60	1.75	1.45	1.71	1.20	1.71	1.20
	SD	0.22	0.25	0.42	0.40	0.43	0.39	SD	0.12	0.24	0.15	0.26	0.16	0.20	0.16	0.20
	*U*	137.50		64.00		61.50		*H*	4.157		8.407		7.784		7.784	
	*p*	0.000 *		0.537		0.331		P	0.041 *		0.004 *		0.005 *		0.005 *	
Relative Advantage	M	1.83	1.58	1.71	1.70	1.88	1.65	M	1.88	1.83	1.89	1.78	1.87	1.84	1.87	1.80
	SD	0.19	0.28	0.22	0.28	0.21	0.26	SD	0.14	0.24	0.18	0.27	0.17	0.22	0.18	0.18
	*U*	129.00		64.00		91.00		*H*	0.031		1.146		0.045		1.389	
	*p*	0.022 *		0.910		0.062		P	0.860		0.284		0.832		0.239	
Compatibility	M	2.00	1.55	2.00	1.75	1.95	1.75	M	1.90	1.84	1.95	1.70	1.89	1.80	1.90	1.72
	SD	0.00	0.37	0.00	.35	0.11	0.38	SD	0.19	0.30	0.12	0.39	0.20	0.45	0.19	0.44
	*U*	36.00		26.00		25.00		*H*	0.096		3.231		0.103		0.679	
	*p*	0.019 *		0.260		0.524		P	0.757		0.072		0.950		0.410	
Observability	M	1.83	1.67	1.91	1.60	2.00	1.62	M	-	-	-	-	-	-	-	-
	SD	0.20	0.33	0.11	0.28	0.00	0.23	SD	-	-	-	-	-	-	-	-
	*U*	30.50		41.00		45.00		*H*	-		-		-		-	
	*p*	0.414		0.038 *		0.001 *		P	-		-		-		-	
Resourcing	M	1.80	1.53	1.89	1.58	1.80	1.62	M	1.96	1.91	1.93	1.89	1.95	1.90	1.94	1.92
	SD	0.14	0.17	0.16	0.16	0.18	0.20	SD	0.15	0.22	0.21	0.22	0.17	0.22	0.17	0.20
	*U*	150.00		119.50		89.00		*H*	0.599		0.227		0.409		0.158	
	*p*	0.000 *		0.001 *		0.083		P	0.439		0.663		0.815		0.691	

Note. Mann–Whitney U is denoted by *U;* Kruskal–Wallis is denoted by *H*. * *p* < 0.05.

## Data Availability

Data is not publicly available due to the confidential nature of the information collected.
